# Method of respiratory rate measurement using a unique wearable platform and an adaptive optical-based approach

**DOI:** 10.1186/s40635-020-00302-6

**Published:** 2020-05-24

**Authors:** Gurpreet Singh, Augustine Tee, Thanawin Trakoolwilaiwan, Aza Taha, Malini Olivo

**Affiliations:** 1 Laboratory of Bio-Optical Imaging, Singapore Bioimaging Consortium, Agency for Science Technology and Research, Singapore, Singapore; 2 Department of Respiratory and Critical Care Medicine, Changi General Hospital, Singapore, Singapore

**Keywords:** Respiratory rate, Automated, Technology, General wards, Breathing pattern, Manual counts

## Abstract

**Background:**

An efficient and accurate method of respiratory rate measurement is still missing in hospital general wards and triage. The goal of this study is to propose a method of respiratory rate measurement that has a potential to be used in general wards, triage, and different hospital settings with comparable performance. We propose a method of respiratory rate measurement that combines a unique wearable platform with an adaptive and optical approach. The optical approach is based on a direct-contact optical diffuse reflectance phenomenon. An adaptive algorithm is developed that computes the first respiratory rate and uses it to select a band. The band then chooses a set of unique optimized parameters in the algorithm to calculate and improve the respiratory rate. We developed a study to compare the proposed method against reference manual counts from 82 patients diagnosed with respiratory diseases.

**Results:**

We found good agreement between the proposed method of respiratory rate measurement and reference manual counts. The performance of the proposed method highlighted deviations with a 95% confidence interval (C.I.) of − 3.34 and 3.67 breaths per minute (bpm) and a mean bias and standard deviation (STD) of 0.05 bpm and 2.56 bpm, respectively.

**Conclusions:**

The performance of the proposed method of respiratory rate measurement is comparable with current manual counting and other respiratory rate devices reported. The method has additional advantages that include ease-of-use, quick setup time, and being mobile for wider clinical use. The proposed method has the potential as a tool to measure respiratory rates in a number of use cases.

## Background

Respiratory rate is one of the most predictive [1–3] and earliest vital signs [4] signaling change in clinical status of patients. Despite this, respiratory rate is also one of the most neglected [5], underutilized, and least recorded [6] vital signs. Several reasons exist for this. Nurses do not often have time, due to heavy workloads and other concerns, to complete a full 60-s measurement by manual counts [5]. Often, a 30-s or 15-s assessment that is multiplied by 2 or 4 is performed that leads to inaccuracies [7, 8]. Poor visibility of the start and end of a breath, interruptions, moving patients, difficulty in counting, or remembering a count can lead to further errors [9].

Technologies to automate respiratory rate measurement can alleviate such issues associated with manual counting. Ginsburg et al. [10] provide an organized and exhaustive list of technologies to measure respiratory rate, grouping them in the way the respiratory rate is measured. Most technologies however are yet to be adopted widely in general care due to their respective limitations. Inductance plethysmography, capnography, piezoelectric, or bioimpedance-based sensors can be used to measure respiratory rate directly. They however can suffer from usability issues, for example, difficulty in getting patients to wear straps around the chest [11]. Acoustic-based sensors can also be used to measure respiratory rate directly. Their performance can however be influenced by environmental noise [12]. Respiratory rate can also be indirectly extracted from electrocardiography (ECG) or photo-plethysmograph (PPG) signals. However, these methods can suffer from accuracy issues despite advancements in signal processing [13].

In this paper, we present a new method of respiratory rate measurement that combines a unique wearable platform for ease of measurement and an accurate and adaptive non-invasive optical approach based on the optical diffuse reflectance phenomenon [14]. The novelty of the presented approach is the use of a unique wearable platform and a non-invasive vertical-cavity surface-emitting laser (VCSEL) driven diffuse reflectance based method, to adaptively and directly measure respiratory rate. The approach uses a VCSEL diode that emits coherent optical radiation on a rest position on chest at micro-Watt emission levels, and uses an integrated photodetector on a second nearby position on chest to sense a diffused collected signal intensity. The stretching of the skin due to thoracic movement results in a net path change and that causes a change in signal intensity at the detector, with a period that corresponds to the respiratory rate. An adaptive signal processing method is used to enhance the device respiratory rate measurements by splitting the signal processing optimizations across different respiratory rate bands.

## Methods

### Setting and participants

A study was designed to benchmark the respiratory rate measure from the proposed method to manual counts (number of breaths per minute). A total of 100 adult inpatients were recruited from the Changi General Hospital, Singapore, between April 11, 2018, and January 16, 2019, in a single arm trial. The Singhealth Centralized Institutional Review Board (IRB) approval was attained for this study (IRB Ref. 2017/2961). The patients recruited were diagnosed with respiratory diseases of asthma, chronic obstructive pulmonary disease (COPD), or pneumonia from the general wards. Out of the 100 patients recruited, 4 patients had data corrupted during monitoring, while 14 patients had unexpected interruptions during all manual counts. The 4 corrupted data were due to saturation in the detected signal. This was remedied for subsequent patients. The 14 patients with interruptions over all manual counts were due to persistent coughing, hence, blocking line-of-sight. Interruptions occurred when another medical staff interrupted ongoing manual counts or when the patients coughed badly to block line-of-sight during manual counts. Such interruptions were noted on case report forms. Eighty-two patients were finally selected for analysis. Minimum sample size was determined using a 1 sample, 2-sided *t* test. A first estimate of mean and standard deviation from 20 volunteers was determined as 1.06 and 2.22, respectively. A second estimate of mean and standard deviation from 56 volunteers at Saw Swee Hock School of Public Health (IRB: S-17-349) was determined as 1.72 and 2.34. This resulted in an upper sample size of 69 for 80% power at.05 significance. Similar sizes were used in [15].

### Protocol

Manual counts were used as reference respiratory rates. To ensure consistency and eliminate variations, a single dedicated and trained medical staff was deployed to observe and manually count respiratory rates. They were taken over 5 min for every patient at 1-min intervals. A total of 4 manual counts per patient were recorded in 5 min. For every patient, electronic and manual recordings were started concurrently. From the electronic recordings, respiratory rates were calculated at the exact 60 s when manual recordings were recorded. They were then benchmarked for comparative analysis. All manual counts and diagnosis were reported on case report forms by hospital medical staff.

### Method of respiratory rate measurement based on VCSEL driven diffuse reflectance and adaptive “sub-banding”

**Fig. 1:**
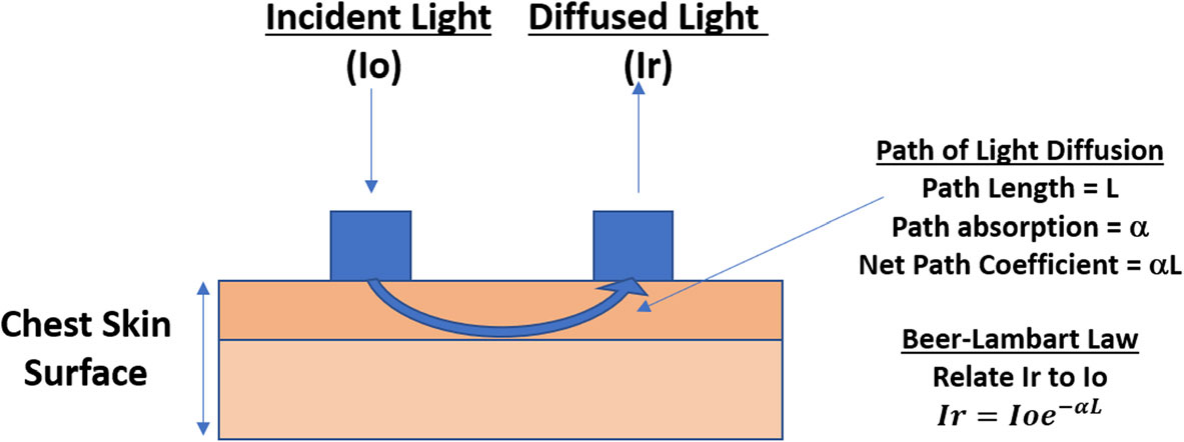
Optical diffuse reflectance approach that is used to extract respiratory rate from the diffused collected signal

The method of respiratory rate measurement combined a unique wearable platform with a VCSEL driven optical diffuse reflectance approach to measure respiratory rate while breathing. The platform emitted a VCSEL-based coherent light onto a rest position on skin (labeled Io in Fig. 1) and collected diffused light from a second position on skin (labeled Ir in Fig. 1). The diffused light consisted of a vibrational component that corresponded to the stretching of the skin. Figure 1 highlights this method.

**Fig. 2:**
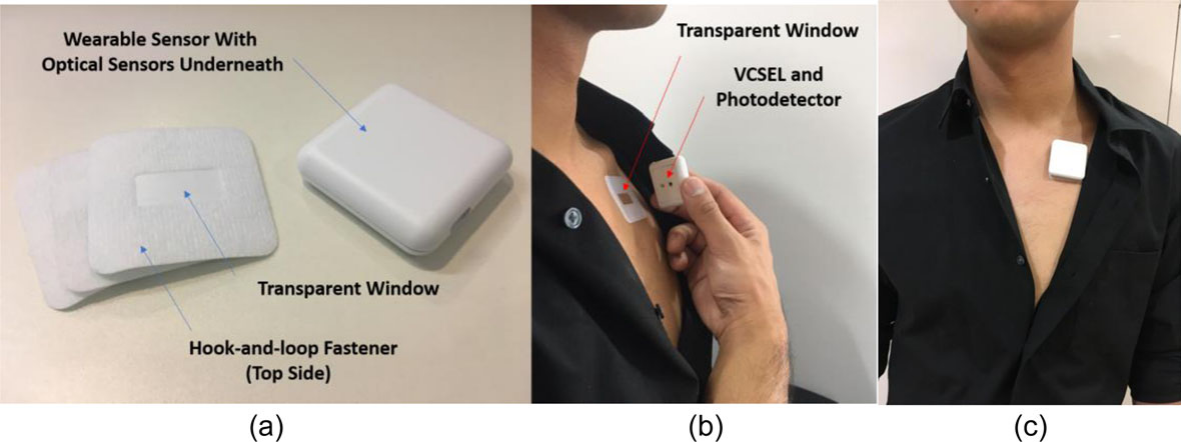
**a** Wearable sensor and patch, **b** side view use of the wearable platform, and **c** front-view use of the wearable platform

**Fig. 3:**
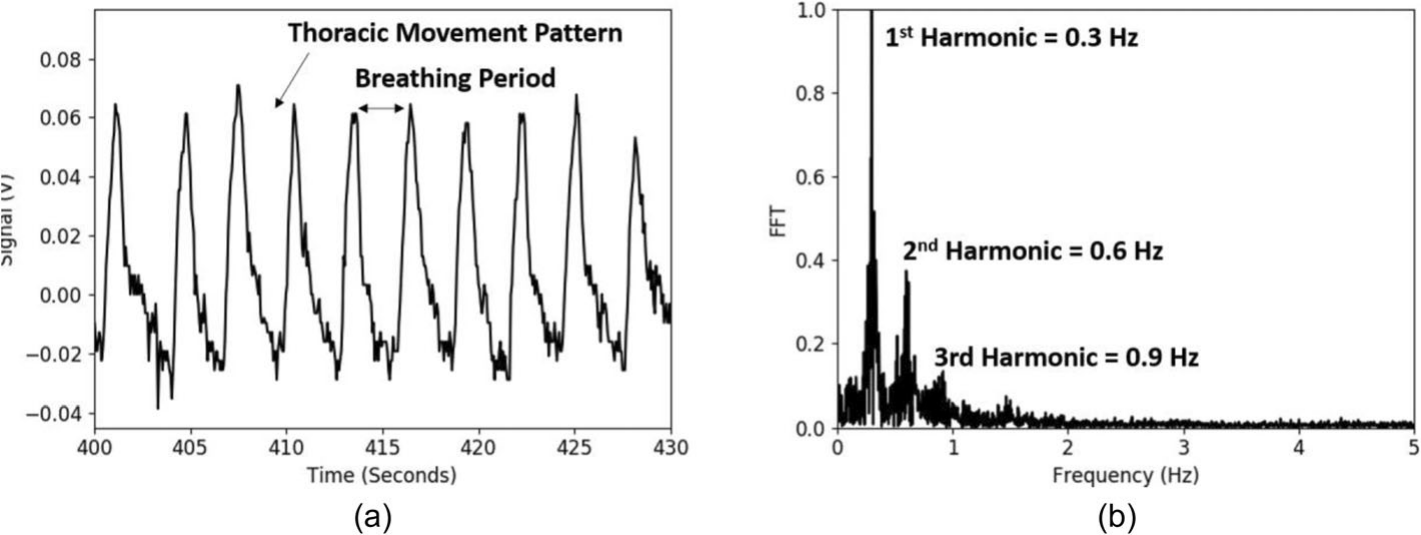
Plots of **a** signal from photo-sensor. **b** Fourier transform showing 1st harmonic that is respiratory rate and the multiple higher-order harmonics

For efficient clinical use, a unique wearable platform design was developed. The wearable consisted of a sensor and a disposable patch. The sensor housed the VCSEL, integrated photo-sensor, microprocessor, and a Bluetooth module. A disposable patch was used to allow light emission from the sensor to be in touch with the skin. One side of the patch was a medical-grade transparent adhesive that stuck to the skin and the other side was a hook-and-loop fastener that connected to the sensor. In the center of the patch was a transparent window that allowed light emission and collection. Figure 2a shows the patch and sensor module that had dimensions of 3.8 cm × 3.8 cm × 1.1 cm and a weight of about 17.3 g. Figure 2b highlights the use of the sensor on a top-left side of a subject’s chest from a side view. Figure 2c shows the front-view use of the device. The average setup time of the device on a subject was 16.1 s. Figure 3a shows an example of the signal intensity at the output of the photo-sensor, after moving average and baseline removal. As can be seen, the signal corresponds to a breathing pattern at a rate equal to the respiratory rate. This breathing pattern was a direct measurement of the thoracic movement. Figure 3b shows the Fourier transform of the signal that verifies the respiratory rate as 0.3 Hz and further shows the higher-order harmonics.

### Analysis

Preprocessing operations were applied to the raw signal before respiratory rate was extracted. A moving average filter was used to remove high-frequency noise and a baseline filter was used to remove any baseline drifts. A motion artifact filter using the Teagar operator [16] was used to identify and correct for motion. Data was then buffered into windows of 60 s, from which, respiratory rate was extracted using a fast time-domain-based algorithm [17].

An adaptive approach was used so as to develop optimized parameters for calculation of respiratory rates based on different respiratory rate bands—an approach labeled as “sub-banding” in this paper. The “sub-banding” approach calculated the first respiratory rate in every window of 60 s, and then determined which range this rate fell in, before applying a second set of optimized parameters to correct the respiratory rate. Hence, each “sub-band” consisted of its own set of optimized parameters. For example, consider there were 3 “sub-bands” defined as 0–15, 16–25, and 26–40 bpm. For each of these “sub-bands,” the parameters of the time-domain-based algorithm [17] that was used to calculate the respiratory rate were uniquely defined. For instance, the number of moving-average filter points (to remove high frequency noise) was defined as 15 points in a lower respiratory band of 0–15 bpm. On the other hand, a lower number of moving-average filter points were defined as 9 in a higher respiratory band of 26–40 bpm. This was to prevent removal of higher frequency components that might have been misinterpreted as noise otherwise. Such optimizations within bands of respiratory rate could improve the quality of the calculation.

Deviations between the device respiratory rate and manual counts were exactly calculated at intervals of 1 min across the 82 subjects. Statistical analysis was then performed on the distribution of deviations with mean, standard deviation, and the 95% confidence intervals extracted.

## Results

The demographics of the study are presented in Table 1.

**Table 1:** Demographics of study

Characteristics	
Male sex (of the 82 included), *n* (%)	45 (54.9%)
Age, year (of the 82 included), mean (SD)	53.7 (16.7)
Patient type (total)	
Total recruited	100
Corrupted data	4
With all manual counts interrupted	14
With all manual counts non-interrupted	64
With at least one manual count non-interrupted	82
Diagnosis (of the 82 included)	
COPD	14
COPD and asthma	5
Asthma	31
Pneumonia	32

**Fig. 4:**
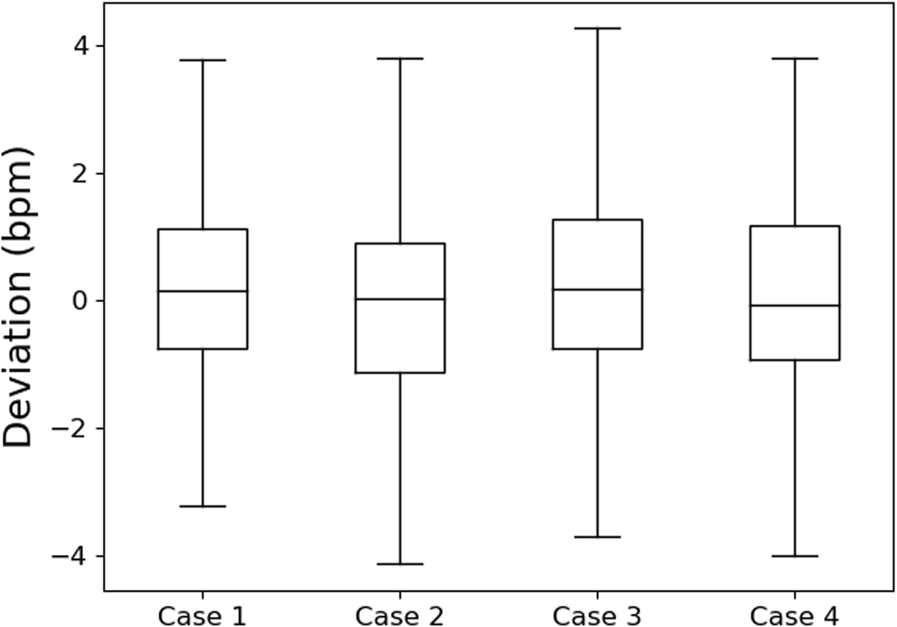
Box plot of deviations between the proposed method of respiratory rate measurement and manual counts, across 4 cases (see Table 2)

Figure 4 is a box plot showing the deviation between the proposed method of respiratory rate measurement and manual counts, in bpm, over 4 different cases. Case 1 corresponds to deviations from only non-interrupted manual counts (64 subjects) and without the adaptive “sub-banding” (normal). Case 2 corresponds to deviations from only non-interrupted manual counts but with adaptive “sub-banding.” Case 3 corresponds to deviations with interrupted manual counts (82 subjects) and without adaptive “sub-banding.” Case 4 corresponds to deviations with interrupted manual counts but with adaptive “sub-banding.” For adaptive “sub-banding,” the bands defined are 0–20, 20–27, and 27–50 bpm. Table 2 highlights these different cases and further notes the mean bias, standard deviation, and 95% confidence intervals of deviations across all 4 cases (in bpm).

**Table 2:** Table highlighting bias, standard deviation, and 95% confidence intervals for the different test cases

Case	Manual count type	Signal processing type	Bias	STD	95% C.I.
1	Exclude interrupts	Normal	0.16	2.38	(− 3.88, 4.40)
2	Exclude interrupts	Adaptive sub-banding	− 0.12	2.34	(− 3.48, 3.40)
3	Include interrupts	Normal	0.17	2.71	(− 3.78, 5.08)
4	Include interrupts	Adaptive sub-banding	0.05	2.56	(− 3.34, 3.67)

**Fig. 5:**
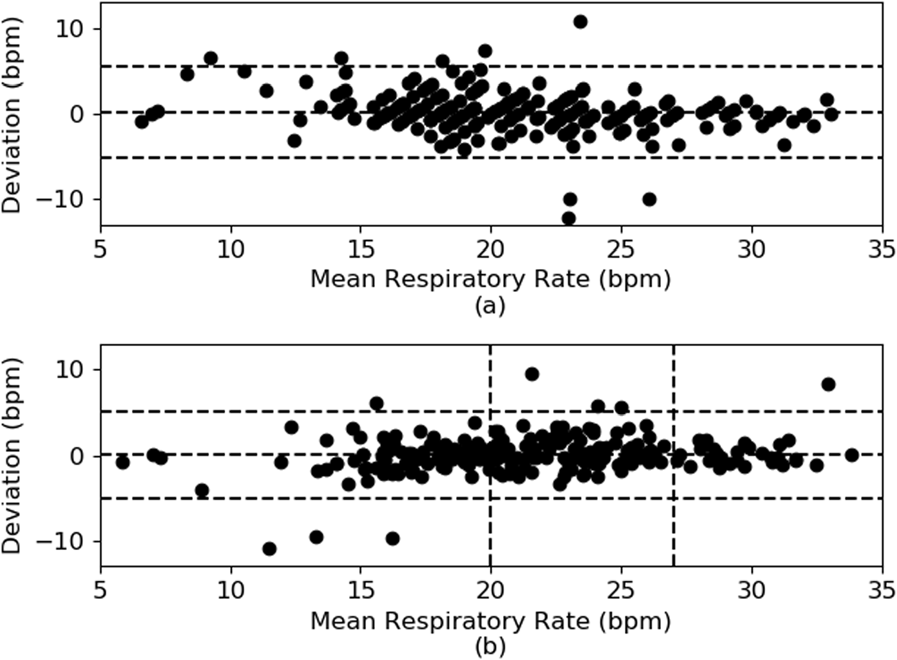
Bland-Altman plots of **a** case 3 (no “sub-banding”) and **b** case 4 (with “sub-banding”)

From the box plot, we can observe that all deviations (across the 4 cases) lie within a box interval of 2 bpm and lie within the whisker interval of 8 bpm across the median that is centered near 0 bpm. From Table 2, we can observe that the amount of deviation increases when interrupt cases are included. We can also observe that the use of adaptive “sub-banding” reduces the deviations. Specifically, with interrupt cases and with the adaptive feature, the 95% confidence intervals lie within the range of − 4–4 bpm. Figure 5 shows the Bland-Altman plots for case 3 and case 4. From these plots, we can observe that the spread in deviation is smaller and more tighter for case 4 (with “sub-banding” algorithm (Fig. 5b)) compared to case 3 (without “sub-banding” algorithm (Fig. 5a)). The sub-bands have been indicated with vertical lines in Fig. 5b. The range of the observed respiratory rate across all patients is 6–41 bpm.

**Fig. 6:**
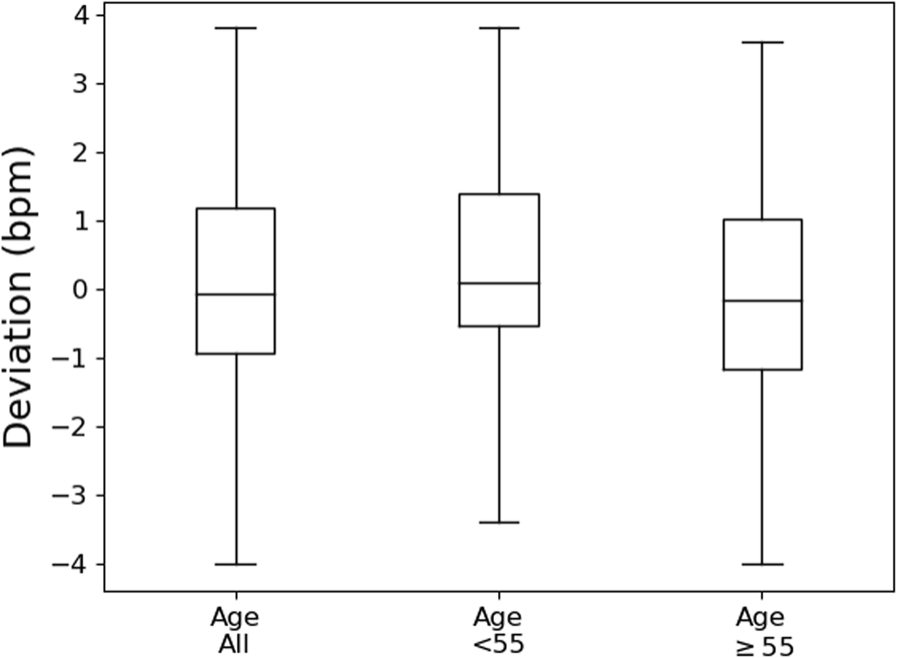
Box plot of deviations between the proposed method of respiratory rate measurement and manual counts for case 4 (see Table 2) and across 2 different age groups

Figure 6 illustrates the performance of the proposed method of respiratory rate measurement using the “sub-banding” algorithm (case 4) across patients of 2 age groups—more than and equal to 55 years of age and patients less than 55 years of age. As can be deduced from Fig. 6, the minimum and maximum whiskers of all age groups, less than 55 years and more than or equal to 55 years, lie within deviations of − 4 and + 4 bpm.

## Discussion

We demonstrated a method of respiratory rate measurement that combined a unique wearable platform and an adaptive optical-based approach. Respiratory rate was directly measured using the device on 82 patients and benchmarked against reference manual counts that were measured by a single dedicated and trained medical staff. An adaptive method that optimized the parameters on the device was also proposed where the initial respiratory rate measure was used to select the band from which a set of optimization parameters was used to correct for the first respiratory rate measure.

We found good agreement between the proposed method of respiratory rate measurement and reference manual counts. The performance of the proposed method highlighted deviations with a 95% confidence interval of − 3.34 and 3.67 bpm and with a mean bias and standard deviation of 0.05 bpm and 2.56 bpm, respectively.

This performance was comparable with performances of other respiratory rate devices reported, as shown in Table 3. The Bland-Altman plots in Fig. 5 further verified the adaptive approach of “sub-banding” in improving the performance of the overall algorithm. There were 5 primary outliers from the Bland-Altman plot of Fig. 5a. The corresponding mean respiratory rates were 23 bpm, 19 bpm, 26 bpm, 23 bpm, and 22 bpm. The corresponding deviations were 10.77, 7.45, − 9.9, − 9.98, and − 12.1, respectively. The 1st and 2nd readings corresponded to patient IDs 7 and 83, while the 3rd, 4th, and 5th readings all corresponded to patient ID 86. Possible reasons for such outliers included coughing for patient IDs 7 and 83, and fast and shallow breathing for patient ID 86.

**Table 3:** Table comparing the performance of other respiratory rate devices reported in literature and the proposed method of respiratory rate measurement

Device	Bias	STD	95% C.I.
Medtronic Nellcor Pulse Oximetry [15]	0.07	1.99	(− 3.84, 3.97)
Masimo RRa [18]	0	1.0	(− 1.9, 1.9)
Nihon impedance pneumography [18]	0.4	5.9	(− 11.1, 11.9)
Proposed method	0.05	2.56	(− 3.34, 3.67)

Bias, standard deviation, and 95% confidence interval are all in bpm

In addition to the proposed method’s performance, the proposed method possessed several other advantages that may be useful for clinical implementation. Firstly, the proposed method used a direct way to measure respiratory rate, unlike methods of PPG or ECG, hence, may require fewer computational steps with direct processing of respiratory rates on sensor. Secondly, the proposed method used an optical-based approach that is potentially less susceptible by environmental noise, compared to, for example, acoustic methods. Furthermore, an optical-based approach may allow the sensor to double up as a pulse oximeter. Thirdly, the proposed method was worn on the chest with quick setup times of average 16.1 s. The sensor module itself can be removed if the patient needs to shower, while leaving the patch on the chest, for the sensor to be replaced for a subsequent set of measurements. The minimum time to read out and analyze data from a mobile platform was 1 min.

The proposed method of respiratory rate measurement did present limitations and some cautionary efforts. Firstly, patients with hairy chest did experience some discomfort when removing the patch from skin. This however was overcome by using thinner, more conformable, hypoallergenic, and less-discomforting types of medical grade patches, some of which had been experimented and verified in this study. Secondly, the sensors were susceptible to motion artifacts. To overcome this, signal processing was implemented to detect and remove/correct for motion artifacts. Thirdly, caution had to be taken not to use the VCSEL light sources at very high powers. These sources are highly coherent and prolong usage at high powers could possibly cause reactions, although VCSELs are well performing and can provide better results. Very low emission levels have thus been used in this study at the order of micro-Watts without any cases of reactions/adverse events over the 100 patients. Fourthly, the present study and method was limited to adult patients. Fifthly, the present study was limited to breathing rates between 6 and 41 bpm. Any impact due to Bluetooth interference was minimal as the device used a low-energy Bluetooth transmitter and satisfied the Federal Communications Commission limit of field strength for medical equipment.

Moving forward, the authors aim to test and explore the proposed method of respiratory rate measurement for several possible use cases. Such use cases include the use of the device for continuous monitoring of respiratory rates (and other breathing features) and to use these rates/features in analytics to better predict deterioration (e.g., COPD exacerbations [19]) in home settings [19–21] and inpatient [22]. The authors also aim to consider the device for other clinical applications that include monitoring sleep [23]. Another possible use case is to use the proposed method to extract newer feature sets from respiratory patterns and to use these feature sets to develop newer classifiers, for example, to quickly screen a patient suffering from COPD or asthma. The authors further aim to expand beyond the adult age group and to test and explore use cases of this device on children, specifically those aged < 16 years, as well as a wider coverage of breathing rates

## Conclusion

We have proposed a method of respiratory rate measurement by combining a unique wearable platform and an accurate and adaptive VCSEL based diffused reflectance approach. The performance of the proposed method of respiratory rate measurement is shown to be comparable to current manual counting for respiratory rate. The performance is also comparable with those of other respiratory rate devices reported in literature. The proposed method has also additional advantages of being easy-to-use, quick to setup, and mobile.

## Data Availability

Datasets used/or analyzed during the current study are available from the corresponding author on reasonable request.

## Abbreviations

ECG: Electrocardiography
PPG: Photo-plethysmograph
bpm: Breaths per minute
VCSEL: Vertical-cavity surface-emitting laser
CIRB: Centralized Institutional Review Board
COPD: Chronic obstructive pulmonary disease
C.I.: Confidence interval
STD: Standard deviation
IRB: Institutional review board
